# Linearity Improvement of MEMS Electrochemical Vibration Sensors Based on Tapered-Hole Technology

**DOI:** 10.3390/mi17030333

**Published:** 2026-03-09

**Authors:** Hongmin Jiang, Honghao Zhang, Wenlang Zhao, Yulan Lu, Deyong Chen, Junbo Wang

**Affiliations:** 1 State Key Laboratory of Transducer Technology, Aerospace Information Research Institute, Chinese Academy of Sciences, Beijing 100190, China; 2 School of Electronic, Electrical and Communication Engineering, University of Chinese Academy of Sciences, Beijing 100049, China

**Keywords:** electrochemical vibration sensor, MEMS electrode, linearity, tapered hole

## Abstract

Electrochemical vibration sensors offer high sensitivity, low mechanical noise, and superior low-frequency performance, making them attractive for applications such as seismic detection and underwater acoustic sensing. However, existing electrochemical seismometers, angular accelerometers, and vector hydrophones primarily focus on sensitivity and noise, while sensor linearity—especially across wide frequency ranges—remains insufficiently investigated. In practice, linearity degradation frequently occurs at low and high frequencies due to diffusion limitations of electroactive species in the electrolyte. In this study, the linearity mechanism of electrochemical vibration sensors is analyzed, and two key structural parameters affecting linearity are identified: one is the anode–cathode spacing and the other is the effective cathode length. To improve linearity, an electrochemical sensing electrode incorporating an ultra-narrow insulating ring and a tapered micro-orifice is proposed. Finite element simulations are performed to evaluate the effects of electrode spacing, orifice geometry and excitation frequency. The sensor is fabricated using MEMS fabrication technology and experimentally characterized. Results show a peak sensitivity of 1242 V/(m/s) and excellent linearity within an input velocity range of 0.0002–0.012 m/s at 5 Hz, 10 Hz, 40 Hz and 100 Hz, with correlation coefficients exceeding 0.998. The proposed design provides an effective approach for linearity enhancement in electrochemical vibration sensors.

## 1. Introduction

Vibration is a ubiquitous physical phenomenon that exists in a wide range of scenarios, including geophysical motion, industrial equipment operation, mechanical and civil structures, and biological rhythms. It represents the inherent dynamic response of physical systems and contains critical information related to structural integrity, operational status, and environmental disturbances [1]. Vibration signals often precede or accompany catastrophic events, failures, or motions, such as early-stage damage in machinery, structural fatigue and cracking, seismic precursors, and underwater target motion [2,3,4,5]. Consequently, the detection and analysis of vibration signals play a vital role in engineering monitoring, fault diagnosis, and environmental sensing.

Vibration sensors are therefore required to convert mechanical vibration signals into quantifiable signals, typically electrical signals, enabling subsequent processing and analysis [3]. Depending on their operating principles, vibration sensors can be categorized into electromagnetic [6,7], capacitive, piezoelectric [8,9], optical fiber-based, MEMS accelerometer, and electrochemical types. Among these, electrochemical vibration sensors (EVSs) employ electrolytes as inertial masses and exhibit several distinctive advantages, including high sensitivity, low mechanical noise, and wide operating bandwidth [4,10]. Owing to these characteristics, EVSs demonstrate unique performance advantages in multiple application domains.

At present, electrochemical vibration sensing has been widely applied in electrochemical seismometers [5,11,12,13,14,15,16], electrochemical angular accelerometers [11,12,13,14,15,16,17,18,19], and electrochemical vector hydrophones [12,13,14,15,16,17,18,19,20,21]. Electrochemical seismometers feature large allowable working tilt angles, enabling reliable operation in complex underground or seabed environments while maintaining sensitivity to ultra-low-frequency signals below 0.1 Hz [13,14]. Electrochemical angular accelerometers achieve extremely low noise levels while preserving high sensitivity and a flat sensitivity response [17,18]. Electrochemical vector hydrophones are capable of effective detection of low-frequency acoustic signals below 20 Hz, overcoming the limitation of conventional vector hydrophones whose operating bandwidths typically exceed 10 Hz [20,21].

Existing studies on electrochemical seismometers, angular accelerometers, and vector hydrophones primarily focus on improving sensitivity, bandwidth, and noise performance. However, the linearity of electrochemical vibration sensors—an equally critical performance metric—has received comparatively little attention. In many reported works, linearity is either omitted or evaluated only at moderate frequencies, typically around 10 Hz. Such characterization is insufficient because the sensing mechanism of electrochemical vibration sensors relies on ion diffusion and convection within the electrolyte. At low frequencies (below 5 Hz) and high frequencies (above 100 Hz), ion transport is constrained by factors such as the anode–cathode spacing and the effective cathode length, leading to linearity degradation. Linearity drift results in sensitivity variation, which hinders accurate reconstruction of vibration amplitude from electrical output signals and consequently limits the practical applicability of electrochemical vibration sensors.

In this study, through theoretical analysis and numerical simulations, two key parameters governing the linearity of electrochemical vector hydrophones are identified: the anode–cathode spacing and the effective cathode length. To improve device linearity, insulating rings and tapered via structures are introduced into the electrode design. The fabricated electrochemical vibration sensor achieves a peak sensitivity of approximately 1242 V/(m/s) and maintains excellent linearity over an input vibration velocity range of 0.0002–0.012 m/s at excitation frequencies of 5 Hz, 10 Hz, and 100 Hz.

## 2. Structure and Working Principle

**Figure 1:**
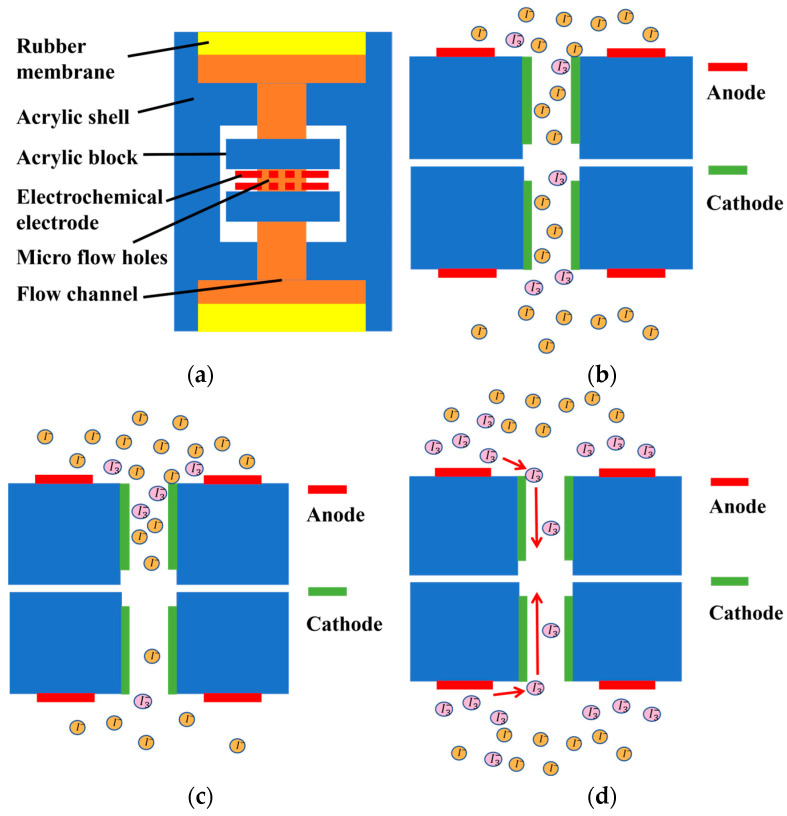
Simplified structure and sensing principle. (**a**) Simplified structure of the electrochemical vibration sensor; (**b**) ion concentration distribution near the electrochemical sensing electrode under static conditions; (**c**) ion concentration distribution near the electrochemical sensing electrode when external vibration is applied; (**d**) generation and motion of $I_{3}^{-}$.

A simplified structure of the electrochemical vibration sensor is illustrated in Figure 1a. The sensor mainly consists of rubber membranes, an acrylic housing, acrylic blocks, and an electrochemical electrode assembly located at the center. The internal cavity is filled with a mixed electrolyte composed of 2 mol/L KI and 0.02 mol/L I_2_. When a bias voltage is applied to the anodes, the following reversible electrochemical reactions occur:

(1) $$\mathrm{Anode}:\;3I^{-}-2e^{-}⟶I_{3}^{-}$$

(2) $$\mathrm{Cathode}:\;I_{3}^{-}+2e^{-}⟶3I^{-}$$

The sensing mechanism of the electrochemical vibration sensor relies on the internal electrochemical electrodes. Under static conditions, the reactions between iodide ions and triiodide ions reach equilibrium, and the ion concentration distributions near the two cathodes are identical, as shown in Figure 1b. After differential signal processing, the output current is zero. When external vibration is applied, the rubber membranes on both sides of the sensor drive the internal electrolyte to oscillate synchronously, converting external vibration velocity into electrolyte flow velocity near the flow channels—this process is referred to as vibration pickup. As the electrolyte oscillates, the ion concentration distributions near the two cathodes change in opposite directions, as shown in Figure 1c, generating a differential current. This process constitutes the mechanoelectrical conversion, also known as the electrochemical transduction process.

The vibration pickup process can be approximated as a damped second-order mass–spring system, where the electrolyte acts as the inertial mass, the rubber membranes provide restoring force, and the flow resistance within the channels contributes to damping [22]. The transfer function of the pick-up process can be expressed as follows [22]:

(3) $$\frac{V_{out}(\omega )}{V_{in}(\omega )}=\frac{\omega^{2}}{\sqrt{{\left(\omega^{2}-\omega_{0}^{2}\right)}^{2}+{Re}^{2}\omega^{2}}}$$

where $\omega_{0}$ is the natural frequency of the system, ω is the excitation frequency, and Re is the flow resistance coefficient determined by the number and geometry of the flow channels. For cylindrical channels, Re satisfies the following [22]:

(4) $$Re∼\frac{1}{nr^{4}}$$

When the packaging structure and rubber membranes remain unchanged, the system’s natural frequency remains constant, and the liquid flow resistance becomes the dominant factor governing the pickup transfer function.

For the electrochemical transduction stage, the cathode current can be derived from Faraday’s law and the Nernst–Planck equation [21,23]:

(5) $$I=qF\int_{s}J\cdot mds$$

(6) $$J=-D\nabla C-\frac{zF}{RT}DC\nabla \varphi +Ckv$$

where *q* is the number of electrons exchanged in the cathodic reaction, F is Faraday’s constant, *s* is the effective cathode area, ***m*** is the normal vector of the cathode surface, and ***J*** is the ion flux. Here, ***v*** denotes the electrolyte velocity near the microelectrodes, C is the ion concentration, *z* is the ionic charge number, R is the gas constant, *T* is the absolute temperature, *φ* is the electric potential, D is the diffusion coefficient, and k is the coefficient characterizing the contribution of convection to the ion flux. Among the three terms in ***J***, diffusion, migration, and convection, the convection term dominates under vibration excitation, meaning ***J*** is proportional to ***v*** [21]. Since obtaining an analytical solution for the electrochemical transduction process is difficult, finite element simulation is adopted as an effective approach.

Because the concentration of iodide ions in the electrolyte is much higher than that of triiodide ions, the cathodic reaction becomes the rate-limiting step of the reversible electrochemical process. Based on the working principles discussed earlier, the mechanism underlying linearity degradation can be analyzed. In the electrochemical vibration sensor, triiodide ions are primarily generated near the anode and diffuse toward the cathode, where electrochemical reactions produce electrical current, as illustrated in Figure 1d.

At low excitation frequencies, the vibration amplitude of ions is relatively large, causing ions to move beyond the effective cathode region. As a result, part of the cathode surface becomes underutilized, leading to linearity degradation. At high excitation frequencies, the vibration amplitude of ions is small, making it difficult for ions to migrate from the anode to the vicinity of the cathode, which also degrades linearity.

Consequently, two key parameters affecting the linearity of electrochemical vibration sensors are identified: the anode–cathode spacing and the effective cathode length. These parameters are systematically investigated through numerical simulations.

## 3. Simulation and Results

### 3.1. Simulation Model

**Figure 2:**
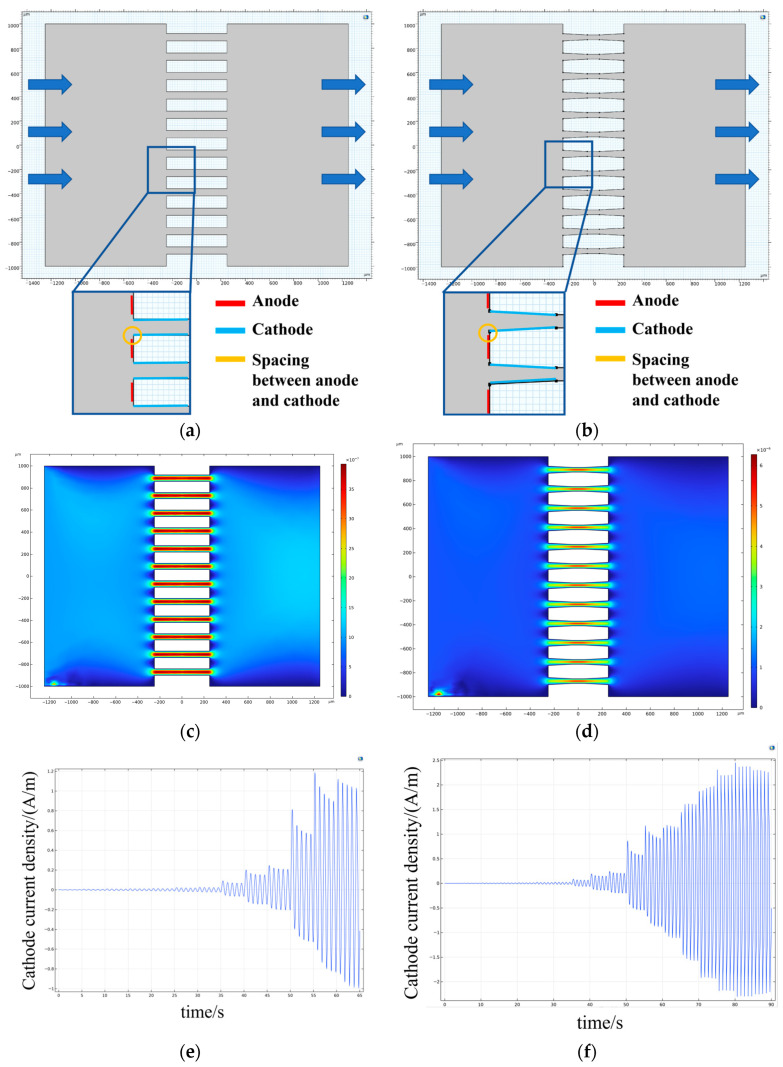
Simulation models. (**a**) Simulation models for electrochemical sensing electrode with cylindrical holes; (**b**) simulation models for electrochemical sensing electrode with tapered holes; (**c**) velocity distribution in the cylindrical flow holes; (**d**) velocity distribution in the tapered flow holes; (**e**) cathode current density of electrochemical sensing electrode with cylindrical flow holes at 1 Hz obtained by integration; (**f**) cathode current density of electrochemical sensing electrode with tapered flow holes at 1 Hz obtained by integration.

Since the transfer function of the vibration pickup process is independent of input velocity amplitude for a fixed chip structure, only the electrochemical transduction stage is simulated. Finite element simulations are conducted to evaluate sensor linearity by varying parameters including anode–cathode spacing, flow channel geometry, cathode deposition depth, input velocity amplitude, and excitation frequency. The simulation models for cylindrical and tapered holes are shown in Figure 2a,b, respectively. Laminar flow and tertiary current distribution physics are employed. The laminar flow inlet is on the left, while the outlet is on the right. A bias voltage of 0.3 V is applied to the anode located on the outer surface of the channel, while the cathode is positioned on the inner channel wall. In a single simulation, a sinusoidal inlet velocity $v(t)=v_{0}sin(2\pi ft)$ is applied on the left side, with the velocity amplitude increasing over time while the frequency f stays the same, and the resulting velocity distribution in flow holes is shown in Figure 2c,d. Cathode current linear density, as the output of the electrochemical sensing electrode shown in Figure 2e,f, is obtained by integrating the cathode current surface density, which is calculated by simulation, over the cathode. By using MATLAB 2023b to calculate the amplitude of cathode current density at different times, we can get the results of the simulation.

### 3.2. Results of Simulation

**Figure 3:**
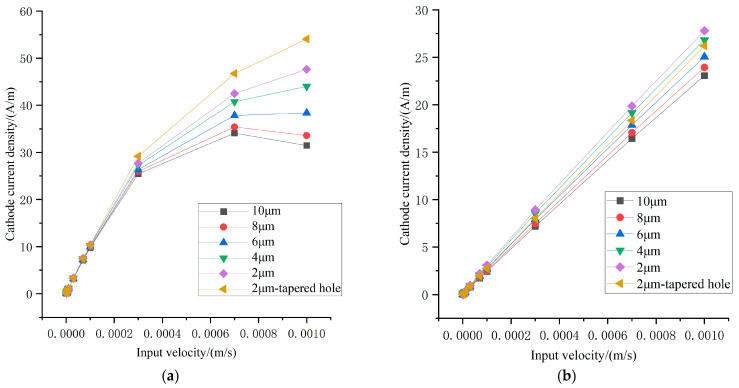

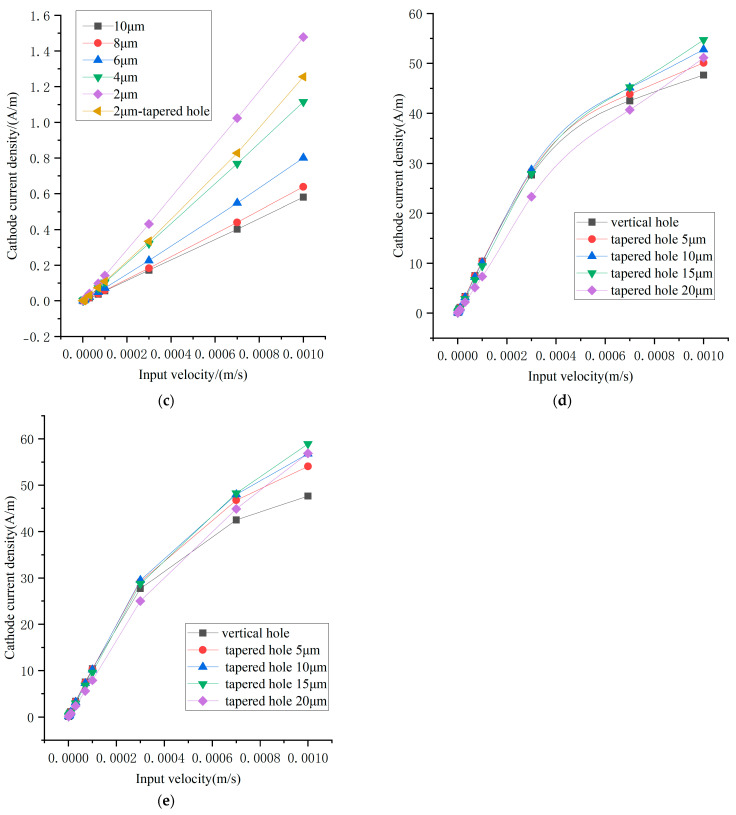
Processed results of simulation. (**a**) Input–output curve when reducing anode–cathode spacing at excitation frequency of 1 Hz; (**b**) input–output curve when reducing anode–cathode spacing at excitation frequency of 10 Hz; (**c**) input–output curve when reducing anode–cathode spacing at excitation frequency of 100 Hz; (**d**) input–output curve when the taper degree of flow holes is changed while maintaining the anode–cathode spacing at 2 μm; (**e**) input–output curve when the taper degree of flow holes is changed while maintaining the anode–cathode spacing at 2 μm and increasing the length of cathode by 10%.

The simulation results are shown in Figure 3a–c, where the input velocity amplitude is increased from 1 × 10^−6^ m/s to 1 × 10^−3^ m/s at excitation frequencies of 1 Hz, 10 Hz, and 100 Hz. At 10 Hz, the sensor exhibits good linearity over the entire velocity range. However, at 1 Hz, output saturation occurs as the input velocity increases, which is undesirable since low-frequency performance is a key advantage of electrochemical vibration sensors. There are two reasons for the output saturation at 1 Hz: firstly, the mechanoelectrical conversion behaves as a low-pass process, meaning its output current is larger at 1 Hz, making saturation more likely to occur. Secondly, at low excitation frequencies, ions easily move beyond the effective cathode region, as discussed in Section 2. The results also show that reducing the anode–cathode spacing significantly improves linearity at low frequencies. Furthermore, replacing cylindrical channels with tapered channels that have a reduced central diameter further enhances linearity, albeit at the cost of slightly reduced sensitivity.

Additional simulations focusing on 1 Hz excitation are presented in Figure 3d,e. As the taper angle increases, sensor linearity improves and saturation decreases markedly. Considering that tapered channels facilitate deeper cathode deposition, increasing the effective cathode length by 10% further enhances linearity.

The underlying mechanisms are summarized as follows. Reducing the anode–cathode spacing promotes ion diffusion from the anode to the cathode, improving high-frequency linearity. Tapered channels enhance linearity through three effects: (i) increasing effective cathode length due to inclined sidewalls and deeper metal deposition; (ii) reducing the ion–cathode distance by contracting flow streamlines under laminar flow conditions and enhance the domination of convection in ***J*** by increasing flow velocity; and (iii) increasing flow resistance, which reduces electrolyte velocity at the channel entrance, shifting the operating point from a nonlinear to a linear region.

## 4. Fabrication and Assembling

**Figure 4:**
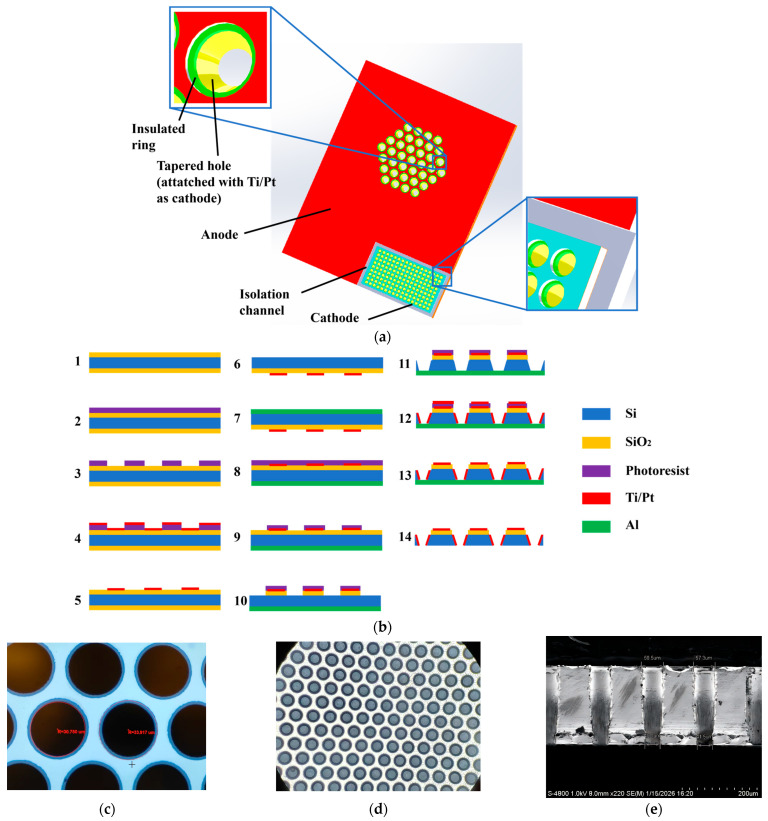

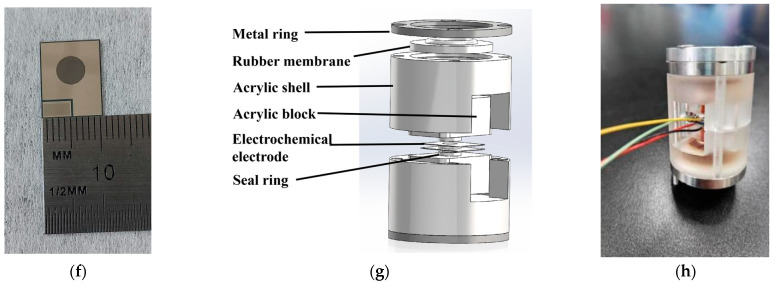
Design, fabrication and assembly of EVS. (**a**) Structure of electrochemical sensing electrode; (**b**) manufacturing process of electrochemical sensing electrode; (**c**) anode–cathode spacing by microscope; (**d**) front view of tapered flow holes by microscope; (**e**) cross-section of tapered flow holes by SEM; (**f**) image of fabricated electrochemical sensing electrode; (**g**) assembly diagram of EVS; (**h**) EVS after assembling.

The structure of a single electrochemical electrode is shown in Figure 4a. The anode is distributed on the electrode surface and biased at 0.3 V, while the cathode is deposited inside the flow channel and electrically connected to the bonding pads through bulk silicon. Two identical electrodes are assembled face-to-face with an insulating rubber ring in between, forming an ACCA (anode-cathode-cathode-anode) electrochemical electrode configuration.

The electrodes are fabricated using MEMS micromachining technology, as illustrated in Figure 4b. The process starts with a 4-inch silicon wafer coated with a 1 μm thermal oxide insulation layer on both sides. After photolithography, Ti/Pt layers are sputtered and lifted off to form the anodes. The backside oxide is then etched, followed by deposition of a 1 μm aluminum layer to protect the wafer during deep reactive-ion etching (DRIE). Flow channels are subsequently patterned and etched using DRIE. The remaining photoresist serves as a mask for sputtering Ti/Pt onto the channel sidewalls to form cathodes. Finally, the aluminum protection layer is removed, completing the fabrication.

Two critical steps affecting linearity are the fabrication of insulating rings and tapered channels. Insulating rings are formed by selectively removing metal at the center of the anode, yielding a narrow insulation region of approximately 3 μm that minimizes anode–cathode spacing, which is shown in Figure 4c. Tapered channels are fabricated by adjusting Bosch process parameters, including cycle times of protecting and etching and gas flow rates of C_4_F_8_ and SF_6_, resulting in channels whose bottom radius is approximately 10 μm smaller than the top radius, which is shown in Figure 4d,e.

The fabricated electrode chip measures 10.6 mm × 13.6 mm, which is shown in Figure 4f. Sensor assembly follows the procedure shown in Figure 4g, involving mechanical fixation, membrane sealing, electrolyte filling, and final hermetic sealing. The physical picture of EVS after assembling is shown in Figure 4h.

## 5. Experimental Characterization

After fixing the EVS and the moving-coil sensor together on the platform [24], they will output an electrical signal when the platform vibrates. We can calculate the vibration velocity of the platform by using the output of a standard moving-coil sensor and further calculate the sensitivity of EVS.

**Figure 5:**
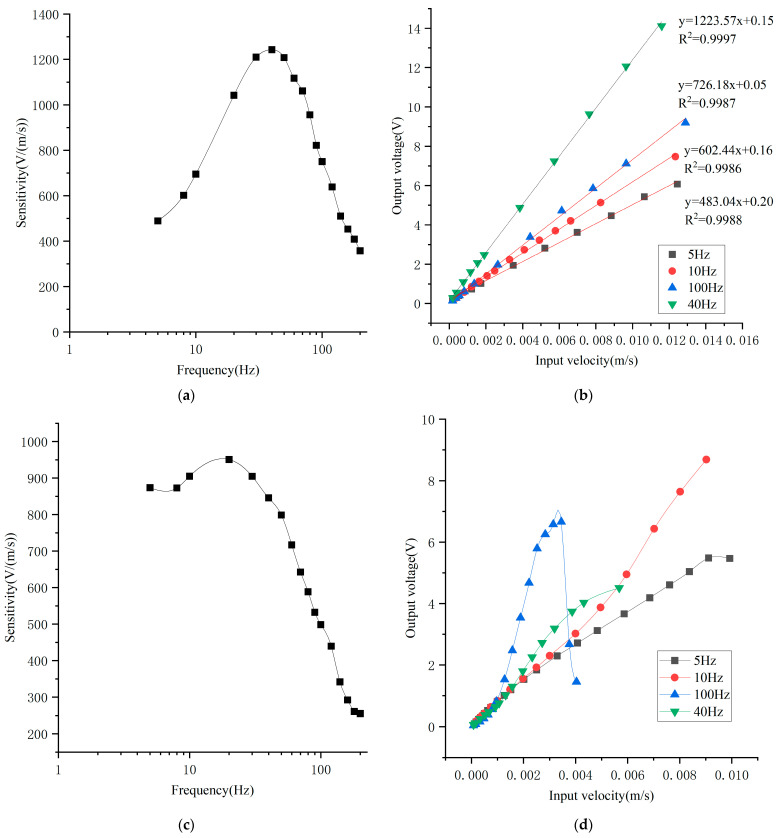
Sensitivity and linearity curve of EVSs. (**a**) Sensitivity curve of fabricated EVS; (**b**) linearity curve of fabricated EVS; (**c**) sensitivity curve of previous EVS [25]; (**d**) linearity curve of previous EVS [25].

Figure 5a shows the sensitivity response, measured by increasing vibration frequency from 5 Hz to 200 Hz while maintaining a constant vibration velocity of 0.3 mm/s. The sensor achieves a peak sensitivity of 1242 V/(m/s) at 40 Hz. Figure 5b presents linearity measurements at 5 Hz, 10 Hz, 40 Hz and 100 Hz. For all three frequencies, the sensor exhibits excellent linearity over an input velocity range of 0.0002–0.012 m/s, with correlation coefficients of 0.9988, 0.9986, 0.9997, and 0.9987, respectively.

For comparison, previously reported electrochemical sensing electrodes [25] are packaged in the same way, meaning the size of EVS, the size of flow channel and the electrolyte concentration are kept the same, and tested under identical conditions—only the electrode is changed. The maximum sensitivity at 20 Hz is approximately 950 V/(m/s), as shown in Figure 5c. Significant linearity drift is observed at 40 Hz and 100 Hz due to larger anode–cathode spacing, while linearity degradation occurs at higher input velocities at 10 Hz, as shown in Figure 5d.

Compared to the previously reported electrochemical sensing electrode [25], the electrode described in this article shows a slight improvement in sensitivity. In terms of linearity, the linearity of the input–output response is enhanced, and the linear operating range is significantly extended. These enhancements in performance are due to improvements in the spacing between the anode and cathode, which is reduced from over 10 μm to 3 μm, and in the shape of flow holes, which is changed from a cylindrical hole to a tapered hole. These improvements make the input–output curve exhibit better linearity. For the previous electrode, when the input velocity is very low, the particles cannot reach the cathode smoothly, such that the input–output curve bends upward; while the input velocity is very high, particles will move beyond the effective cathode region, leading to linearity degradation. Thus, the input–output curve of the previous electrode exhibits an “S-shaped” characteristic, which is more obvious at a high input frequency. For an improved electrode, reducing the spacing between the anode and cathode can enhance particle diffusion between the electrodes, while the tapered flow hole can mitigate input saturation and improve the linear relationship between the output and the input. As a result, the electrode proposed in this article exhibits good linearity.

A comparison of the key parameters of the different electrochemical vibration sensors is shown in Table 1, which indicates that the EVSs fabricated in this article have relatively high sensitivity and excellent linearity across wide frequency ranges.

## 6. Conclusions

In this study, the linearity limitations of conventional electrochemical sensing electrodes are systematically analyzed. A novel electrochemical sensing electrode featuring tapered flow holes is designed, simulated, fabricated, and experimentally characterized. The fabricated electrode achieves an anode–cathode spacing of approximately 3 μm and employs tapered holes with a radius decreasing from 30 μm to 20 μm. The sensor maintains excellent linearity over an input velocity range of 0.0002–0.012 m/s at excitation frequencies of 5 Hz, 10 Hz, 40 Hz and 100 Hz, while achieving a peak sensitivity of 1242 V/(m/s).

This work represents the first application of DRIE-fabricated tapered flow holes in electrochemical sensing electrodes, simultaneously increasing effective cathode length, enhancing flow resistance, and improving linearity. The proposed approach provides a new pathway for electrochemical electrode design and is expected to benefit the development of electrochemical vector hydrophones and seismometers, enabling improved linearity while preserving high sensitivity for more accurate detection of seismic and hydroacoustic vibration signals.

## Figures and Tables

**Table 1:** Comparison of key parameters of electrochemical vibration sensors.

Characteristics	Unit	[25] (Measured)	[20]	[26]	This Article (Measured)
sensitivity	V/(m/s)	950@10 Hz	563.44@80 Hz	3369@20 Hz	1242@40 Hz
linearity scale@5 Hz	m/s	0.0002–0.003	not mentioned	not mentioned	0.0002–0.012
linearity scale@10 Hz	m/s	0.0002–0.004	0.0002–0.013	not mentioned	0.0002–0.012
linearity scale@40 Hz	m/s	0.0002–0.0016	not mentioned	not mentioned	0.0002–0.012
linearity scale@100 Hz	m/s	0.0002–0.0004	not mentioned	not mentioned	0.0002–0.012

## Data Availability

Data available on request from the authors.
